# Debating DSM-5: diagnosis and the sociology of critique

**DOI:** 10.1136/medethics-2013-101762

**Published:** 2013-12-10

**Authors:** Martyn D Pickersgill

**Keywords:** Clinical Ethics, Psychiatry, Sociology, Concept of Mental Health

## Abstract

The development of the fifth edition of the American Psychiatric Association's *Diagnostic and Statistical Manual of Mental Disorders*—the DSM-5—has reenergised and driven further forward critical discourse about the place and role of diagnosis in mental health. The DSM-5 has attracted considerable criticism, not least about its role in processes of medicalisation. This paper suggests the need for a sociology of psychiatric critique. Sociological analysis can help map fields of contention, and cast fresh light on the assumptions and nuances of debate around the DSM-5; it underscores the importance of diagnosis to the governance of social and clinical life, as well as the wider discourses critical commentaries connect with and are activated by. More normatively, a sociology of critique can indicate which interests and values are structuring the dialogues being articulated, and just how diverse clinical opinion regarding the DSM can actually be. This has implications for the considerations of health services and policy decision-makers who might look to such debates for guidance.

## Introduction

The fifth edition of the American Psychiatric Association (APA) handbook of diagnosis, the *Diagnostic and Statistical Manual of Mental Disorders* (DSM-5), was published in spring 2013. Readers will not, however, be encountering much of its content for the first time. For several years now, the substance of the DSM-5 has been poured over and debated, released online for scrutiny and comment, and in the process subjected to wide-ranging contestation and critique. Here, I consider some of these critical comments. My intention is not to wade into and further populate an already crowded field of debate. Rather, I aim to underscore the import of a ‘sociology of critique’; that is, an exploration and analysis of the kinds of critical engagements evident within discourse associated with a particular field, and an attention to who is making these, how, why and with what effects. This includes unearthing some of the assumptions and wider debates associated with the various criticisms of DSM-5. Focusing on critique implicitly reiterates a useful sociological point: there is not ‘a’ viewpoint within the mental health professions regarding the DSM that all clinicians necessarily and unproblematically align themselves with.

This essay draws on materials collected as part of broader sensitising and contextualising work being carried out as part of a Wellcome Trust-funded Fellowship around the social ethical dimensions of mental health (and hence does not present empirical findings from a specific and defined research work-package per se). To this end, I begin by noting some of the key concerns felt by many (and advanced in biomedical literature and popular media) with regard to the new diagnostic manual. The article then moves to unpick the specific criticisms made by Thomas Insel, Director of the US National Institutes of Mental Health (NIMH)—a powerful public sponsor of mental health research. These have attracted considerable attention. In order to underscore how critical mental health professionals themselves can be of the infrastructure governing their work, I have drawn especially on the critique emanating from psychiatric and psychological practitioners. I have selected these comments on the basis of how regularly I encounter them in my discussions about mental health with professionals, patients and wider public.

The depth of feeling inherent to some of the appraisals to be explored more fully below should not, perhaps, surprise us. As Annemarie Jutel rightly states in her sociological treatise on diagnosis: ‘The power of diagnosis is remarkable’.^1^ Diagnosis is a means of focusing clinical attention, indicating treatment, suggesting prognosis, and sometimes conferring social and economic benefits. My own attention to diagnosis is shaped by a broadly social constructionist engagement with mental health and illness in that I view diagnostic categories as being constituted, in part, through professional, patient and political claims-making and debate.^2^ In the idiom of philosopher Ian Hacking, I take diagnosis to be an apparatus through which individuals ‘make up’ themselves and one another.^3^ Through this, individuals and societies learn how to recognise ‘normal’ and ‘pathological’ experiences, relate to substances and practices (such as pharmaceuticals and psychological therapies), and negotiate situations saturated with moral feeling and implications (restraint, discharge and access to services being just a few examples).^4–9^ In this sense, diagnostic texts like the DSM come to shape, and be shaped by, a wide range of social actors and institutions, and hence have salience for these—perhaps especially in the case of more contested categories.^10^ ^11^

## The controversy over DSM-5

In September 1999, the APA held its first conference—jointly sponsored by the NIMH—on the ‘DSM-V’ (at this point, Roman numerals were being used in the acronym for the proposed new edition of the manual). This event sought to set research priorities relevant for future classifications, rather than explore the necessary steps for making a new manual.^12^ Since then, the APA has been a hive of activity with regard to the development of its new nosology. The DSM Task Force has generated considerable, and sometimes acrimonious, debate. Criticism has come from a range of quarters within the USA and UK, perhaps most prominently from many leading members of the biomedical community itself.

In February 2010—4 years after the APA appointed David Kupfer (University of Pittsburgh) to Chair the DSM-5 Task Force—the website http://www.dsm5.org presented draft criteria for the new manual. This has subsequently been framed by the APA as an attempt to democratise processes of diagnostic innovation. However, this only occurred after broad and serious concerns were advanced by a number of psychiatrists that key decisions were being made ‘behind closed doors’.^13^ ^14^ Following the publication of the draft, various articles and commentaries were produced—many focusing on the main alterations that Kupfer's committee was endeavouring to make to the DSM. These included a number of major diagnostic changes (although subsequently some of these decisions were overturned).^15^ Thereafter, a wave of criticism washed over the Task Force.

At the most general level, this was directed at the so-called medicalisation of normality that the DSM-5 was deemed to be driving. This refers to the mechanisms by which bodily and psychological experiences come to be situated within and understood through a medical framework.^16^ As sociologist Nikolas Rose points out, ascribing a process as one of medicalisation immediately casts it as ‘suspect’.^17^ In a much discussed editorial, Til Wykes and Felicity Callard noted that a range of new categories were proposed for introduction in the new manual and hence the doubts about its validity multiplied.^18^ Allen Frances, Chair of the DSM-IV Task Force, came to be a remarkably prolific and vocal critic of the proposed changes. In a *BMJ* editorial, he described the ‘grave’ consequences of ‘false positive epidemics’ of disorders that would be constituted through inappropriate usage of new diagnostic entities; in so doing, DSM-5 would ‘expand the territory of mental disorder and thin the ranks of the normal’.^19^ In other words, it would help to further ‘medicalise’ society.

Such points were reiterated by Frances across a number of platforms, including interviews with the media, a blog for the US magazine *Psychology Today*, academic articles and two books.^20^ ^21^ That they found a home in general readership forums like *Psychology Today* could, perhaps, suggest a degree of resonance between Frances’ position and wider public feeling regarding the medicalisation of different kinds of experiences recognised by many as ‘normal’. However, social scientists Allan V Horwitz and Jerome C Wakefield nevertheless argue persuasively that US citizens have come to be increasingly accepting of medication for DSM disorders (which we might infer as indicating at least *some* public acceptance of their legitimacy).^22^ This speaks to the complex relationships that a variety of actors have with the DSM, and underlines diversity of positions that can be maintained regarding the DSM in professional and public debate.

Alongside and related to concerns around medicalisation per se were specific criticisms centring on particular categories. The incorporation of Asperger's Syndrome within Autism Spectrum Disorder is one example; concerns here included the implications for longitudinal research following diagnostic shift, and the effects of change on lobbying, support and services by and for individuals previously labelled with Asperger's Syndrome.^23^ The role of grief within Major Depressive Disorder formed another locus of debate (and has been written about extensively by Frances). This is of particular interest here as a consequence of its relationship with wider arguments about the DSM and the extent to which it was incorporating ever more aspects of, for some, everyday experience within a psychiatric rubric.

To briefly summarise, the DSM-IV included a ‘bereavement exclusion’ aimed at parsing out depression following grief that could be regarded as normal from that which might indicate mental illness. Specifically, it was advised that major depression not be diagnosed in individuals within 2 months of the death of a loved-one. However, the DSM-5 Task Force removed this from the new manual, justified in terms of bereavement not necessarily being an excluding factor for clinically-salient depression, and indeed potentially a precipitating factor to it. This elicited a hostile response from clinicians, patients and activists, and other publics.^24^ Such concerns were highlighted in various news media (especially in the USA and the UK), including the *Huffington Post*, the *Jerusalem Post*, the *Sydney Morning Herald*, *USA Today* and *Wired*. This wider discussion, developed from a specific professional debate, highlights the great degree to which a range of actors in Anglophonic countries where the DSM has traction find (or, at least, are assumed by journalists to find) the idea that grief could potentially be classed as a mental disorder highly unpalatable. Consequently, this case also indicates the ‘lines in the sand’ that can, sometimes, be deemed possible to draw around the jurisdiction of medical authority.

In some senses, there is nothing especially novel about the debates noted above: critics have long attacked the validity and reliability of the DSM, and indeed the wider kinds of medicalisation it is often deemed to promote.^25^ Relating to this are criticisms of the role of drugs for mental health conditions. The pharmaceutical industry can be regarded as what Jutel calls an ‘engine of diagnosis’, which helps to power changes to the APA nosology and then becomes further embroiled in the processes of medicalisation that help to support ‘pharmaceuticalisation’.^26^ This is the tendency to reframe (and perhaps ‘create’) pathologies as amenable to pharmaceutical intervention, which some find disquieting.^27^ Accordingly, we can see how criticisms of the DSM-5 can be better understood when viewed as part of a wider tradition of psychiatric critique, as well as more recent forms of contestation around biomedicine. This history of critique provides a seed bed for contemporary concerns to grow and flower, as well as a structural logic for their articulation (ie, a sense of how, in what format and where they should be advanced in order to be heard). At the same time, the debate the DSM-5 propels helps to bring these longstanding issues more sharply into focus.

## The APA and the NIMH

A major point of departure in the wider debate around DSM-5, (re)contouring the topology of critique, was the apparent distancing of the US NIMH from this text. On 29 April 2013, NIMH Director Thomas R Insel published, as part of his regular ‘Director's Blog’ series, a riposte to the APA manual.^28^ Starting by acknowledging some of the contention associated with DSM-5, and the kinds of changes that might be made, Insel went on to say:

The strength of each of the editions of DSM has been “reliability” […]. The weakness is its lack of validity. Unlike our definitions of ischemic heart disease, lymphoma, or AIDS, the DSM diagnoses are based on a consensus about clusters of clinical symptoms, not any objective laboratory measure. In the rest of medicine, this would be equivalent to creating diagnostic systems based on the nature of chest pain or the quality of fever.^28^

More boldly still, Insel noted that this ‘is why NIMH will be re-orientating its research away from DSM categories’.^28^

Insel's piece attracted considerable attention, with key US (and other international) news media picking it up (eg, the *Globe and Mail*, the *Guardian*, the *Huffington Post*, *Time* and the *New York Times*). That such an august research institution as the NIMH seemed to be attacking—or, at the very least, questioning—the DSM appeared to legitimate wider and ongoing debate about the place, role and impact of diagnosis. As a sociologist of mental health, over summer 2013 it was common for people (eg, patients, professionals, other interested publics) to ask me whether the NIMH was moving away from the DSM, and the implications of this for the validity of the text and the diagnostic categories it contained.

It is not clear, however, that Insel had any intention of attacking a wider project of diagnosis for mental ill-health. In particular, his blog highlighted the ‘Research Domain Criteria’ (RDoC) initiative. This aims to define ‘basic dimensions of functioning’ which could ‘be studied across multiple units of analysis, from genes to neural circuits to behaviors, cutting across disorders as traditionally defined’, with the intent of increasing innovation for the treatment of mental disorders.^29^ The RDoC can be seen as part of a broader ‘technosomatic emphasis’ long evident within US psychiatry and the NIMH (ie, a concentration on bodily correlates of mental ill-health, and the use of technologies to render them more visible to scientists and clinicians).^11^

The RDoC initiative also articulates with wider investigative efforts around the location of ‘biomarkers’ for health and illness.^30^ In so doing, it represents a form of ‘biomedicalisation’; that is, a form of medicalisation that is intrinsically linked with specialised biological science focusing on bodily processes and signs of pathology that operate at a level often too microscopic for inspection within standard clinical settings.^31^ Insel's blog makes plain these links to developments around biomarkers in other areas of medicine:

Some will see RDoC as an academic exercise divorced from clinical practice. But patients and families should welcome this change as a first step towards “precision medicine,” the movement that has transformed cancer diagnosis and treatment. RDoC is nothing less than a plan to transform clinical practice by bringing a new generation of research to inform how we diagnose and treat mental disorders.^28^

While Insel is clearly critical of DSM-5, his main purpose in contributing to claims-making about this manual is to use his critique as a vehicle for advancing a specifically NIMH research agenda, taking advantage of the enlargement of the discursive sphere around mental health that the advent of the DSM enjoined in order to do this. This same strategy had been employed previously, in 2010, when the journal *Science* published a news piece on the development of the RDoC and its relationship to the APA manual a month after the DSM-5 draft criteria had been published online.^32^ In light of the attention Insel commanded, it is thus notable that the RDoC project was not unveiled in tandem with the DSM-5 but rather had been progressing for several years. Indeed, the emphasis of RDoC on the biology of psychopathology will no doubt be troubling to many who construe Insel's blog as being a larger attack than I suggest it should be interpreted as being.

This reading makes more sense when we examine the subsequent press release produced by the NIMH on the 13 May 2013, and jointly authored by Insel and Columbia University's Jeffrey A Lieberman, President-elect on the APA. In it, we can read that contrary to any notion that Insel was espousing anything like ‘antipsychiatric’ ideas, the NIMH and APA have (as the title of the press release puts it) ‘shared interests’:

Today, the […] [DSM] along with the International Classification of Diseases (ICD) represents the best information currently available for clinical diagnosis of mental disorders. Patients, families, and insurers can be confident that effective treatments are available and that the DSM is the key resource for delivering the best available care. The […] [NIMH] has not changed its position on DSM-5.^33^

The press release concludes by noting that ‘By continuing to work together, our two organizations are committed to improving outcomes for people with some of the most disabling disorders in all of medicine’.^33^

Insel's comments, the media interest surrounding them and the swift attempt to dispel this are revealing of the multiple subject positions, hopes, expectations and concerns that circulate around the projects of diagnosis in mental health. To begin with, Insel's blog post illuminates the ambivalence the DSM often elicits. For instance, it is not uncommon for health professionals to use this manual while simultaneously feeling uneasy about its contents.^34^ Insel did not say anything particularly new; the significance of his comments rather lies in the extent that they were brought into public discourse. While Insel's reflections were rendered newsworthy as a consequence of his office and the attention the DSM was receiving at the time, it is unclear whether wider debates were reshaped in the process.

In particular, the focusing of some wider discussion about Insel's blog on his perceived rejection of the DSM, as opposed to his espousing of an NIMH alternative, served to problematise one particular diagnostic enterprise within psychiatry, rather than encouraging debate about diagnosis per se. Moreover, it also elided the clinical and ethical implications of Insel's own biomedical focus and the ever-more somatic approach the RDoC appears to seek to propel (which resonates with the biological emphasis evident in US psychiatry and the APA). The discursive field within which criticisms of the DSM were voiced was thus expanded by Insel, but its nature did not appear to be remade.

Nevertheless, the rapidity with which the NIMH and APA sought to close down the public debate via the release of a joint press release casts a bright light on two key issues. First, the potential reputational capital lost for the APA, its need to regain this and its power in being able to make this public reclamation. Second, the profound importance of diagnosis in general and the DSM-5 per se in the administration of pathology, in terms of treatment, insurance, research and the management of daily life for very many people. Accordingly, public doubts about DSM-5 were, it seems, held to be deeply troubling by both the NIMH and APA, and a clear statement about its current utility necessarily advanced in order to uphold trust and confidence in both diagnostic categories and (US) psychiatry more broadly. The engines of diagnosis, then, continue to turn.

## Discussion

The DSM-5 has attracted broad criticism. This critique has a ‘social life’, and sociological engagement with that reveals both the underlying assumptions critical commentary rests upon as well as which forms produce most traction. Appraisals of the DSM-5 which take as their focus the capacity of this handbook to become embroiled in processes of medicalisation are especially prevalent, and have been made by many mental health professionals themselves (as well as by activists and other ‘stakeholders’, evidenced through interviews in and responses to some of the news media noted above). Such a critique does not represent a radical departure from established critical commentary on psychiatry and psychology.^25^ Rather, we might see the anticipatory discourse stimulated by the advent of DSM-5 as reenergising longstanding debates around the utility and validity of the APA nosology.

News media and other kinds of online commentaries have circulated around already active groups and actors with a history of providing robust commentary on the DSM (in particular, see the many posts on and linking from/to journalist Robert Whittaker's website—http://www.madinamerica.com—which is one important umbrella site for critical engagements with psychiatry). However, within media items themselves, the critique foregrounded has commonly come from already prominent clinicians and scientists, as well as third-sector organisations with a considerable public presence (eg, the mental health charity, Mind). Contributions from smaller support groups, and individual activists and patients, are less visible. This should not necessarily surprise us—and, moreover, there are instances of media coverage situating debates about the DSM in a wider clinical and social context (take, for instance, the wide-ranging coverage of the DSM to be found in the web pages of the *Guardian* newspaper). Yet, we might query whether the inclusion of non-clinical voices and perspectives would have occurred at all if it was not for key psychiatrists such as Allen Frances questioning the tools of their own profession.

Furthermore, focusing primarily on the kinds of criticisms made by Frances—that is, regarding whether particular diagnostic categories are valid or reliable—also risks distracting us from other kinds of critical engagement. Specifically, we might usefully consider whether scholars and other commentators on the DSM could broaden out their arguments over whether diagnostic entities contained within the DSM are ‘right’ or ‘wrong’—or even ‘good’ or ‘bad’—and reflect more fully on the kinds of rights and responsibilities that different diagnoses and diagnostic tools enable and constrain.^5^ ^35^ ^36^ By this I mean, for instance, where and how diagnoses are used both to facilitate and exclude individuals from accessing particular services and benefits, and indeed how they are employed in the actual design of services. This perspective recognises just how constitutive practices of diagnosis are to psychiatry, psychology and nursing, while also recalling that mental health professionals can view these antagonistically, or as a kind of ‘convenient fiction’ to enable communication, collaboration and care.^34^ A renewed focus on the uses to which diagnostic categories are put—and an expansion of the sphere of public discourse around this to include voices which are less seldom heard—might provide a more practice-orientated and potentially patient-centred basis for normative assertions about the design and delivery of mental health systems.

To be clear: I am not claiming that arguments over the capacity of diagnoses to, for instance, form the basis for the medicalisation of ‘normal’ behaviour are redundant. Critical work which carefully and clearly interrogates the making and meaning of diagnostic categories remains vital, and contributes to wider social and clinical debates that remake the realities they emerge from. Nor, indeed, do I wish to suggest that that much important work on the use of diagnosis has not already been carried out (and perhaps especially by service user organisations in the form of evaluation, research and resources). Diagnostic entities are powerful things, shaping society and individual subjectivities; it is, then, precisely for this reason that we should concern ourselves with what they *are* and with what they *do*.

## Conclusions

The development of DSM-5 has resulted in wide-ranging critical commentary, underscoring the importance of diagnosis to the governance of social life. A sociological analysis of critique can help map out its multiple dimensions, as well enable us to understand and interrogate the assumptions it is built around and the uses to which it is put.^37^ In so doing, an analysis of a critique of one specific case—that is, the DSM—can reveal how debate is contoured by a broader ‘ecology’ of criticism associated with the field (ie, mental health) more generally (eg, concerns around pharmaceuticalisation and medicalisation). This in itself has normative implications since it directs attention to concerns and issues that are not being so loudly voiced, and voices which are not allowed to be heard. For biomedical ethics, it also enjoins a sensitivity to nuance and the contexts within which knowledge claims are advanced, especially when these are heavily inflected with moral discourse (eg, regarding the validity of changes to the category of depression). Attending to the plurality of perspectives different actors and institutions express in relation to diagnostic entities, tools and practices reminds us that uniformity of opinion within professional (but also patient and other) communities cannot be taken for granted. This must necessarily be kept in mind when formulating ethical questions and directives that involve diagnosis and DSM-5.
